# A Decision Aide for the Risk Stratification of GU Cancer Patients at Risk of SARS-CoV-2 Infection, COVID-19 Related Hospitalization, Intubation, and Mortality

**DOI:** 10.3390/jcm9092799

**Published:** 2020-08-30

**Authors:** Dara J. Lundon, Brian D. Kelly, Devki Shukla, Damien M. Bolton, Peter Wiklund, Ash Tewari

**Affiliations:** 1 Department of Urology, Icahn School of Medicine, Mount Sinai Hospitals, New York, NY 10029, USA; dara.lundon@mountsinai.org (D.J.L.); d.shukla@mountsinai.org (D.S.); p.wiklund@mountsinai.org (P.W.); 2 Department of Urology, Austin Health, Melbourne, VIC 3084, Australia; drbriankelly@gmail.com (B.D.K.); damienmbolton@gmail.com (D.M.B.)

**Keywords:** COVID-19, risk calculator, urologic oncology, genito-urinary cancer, mortality, decision curve analysis

## Abstract

Treatment decisions for both early and advanced genitourinary (GU) malignancies take into account the risk of dying from the malignancy as well as the risk of death due to other causes such as other co-morbidities. COVID-19 is a new additional and immediate risk to a patient’s morbidity and mortality and there is a need for an accurate assessment as to the potential impact on of this syndrome on GU cancer patients. The aim of this work was to develop a risk tool to identify GU cancer patients at risk of diagnosis, hospitalization, intubation, and mortality from COVID-19. A retrospective case showed a series of GU cancer patients screened for COVID-19 across the Mount Sinai Health System (MSHS). Four hundred eighty-four had a GU malignancy and 149 tested positive for SARS-CoV-2. Demographic and clinical variables of >38,000 patients were available in the institutional database and were utilized to develop decision aides to predict a positive SARS-CoV-2 test, as well as COVID-19-related hospitalization, intubation, and death. A risk tool was developed using a combination of machine learning methods and utilized BMI, temperature, heart rate, respiratory rate, blood pressure, and oxygen saturation. The risk tool for predicting a diagnosis of SARS-CoV-2 had an AUC of 0.83, predicting hospitalization for management of COVID-19 had an AUC of 0.95, predicting patients requiring intubation had an AUC of 0.97, and for predicting COVID-19-related death, the risk tool had an AUC of 0.79. The models had an acceptable calibration and provided a superior net benefit over other common strategies across the entire range of threshold probabilities.

## 1. Introduction

The characteristics of those most at risk of adverse outcome in COVID-19 include male gender, more advanced age, and co-morbidities such as hypertension and diabetes [1,2], risk factors shared with those at greatest risk of a urologic cancer [3]. There are further shared risk factors as the pathogenesis of SARS-CoV-2 infection is mediated in part by angiotensin-converting enzyme 2 (ACE-2) and transmembrane protease serine 2 (TMPRSS2). SARS-CoV-2 host cell entry is facilitated by viral spike proteins, primed by TMPRSS2-mediated cleavage, which bind to ACE-2 and gain access [4]. ACE2 receptors are highly expressed in cells of the bronchial epithelium, alveoli (type 2 cells), myocardium, GI tract, renal proximal tubule epithelium, and bladder epithelium, suggesting that SARS-Cov-2 infection not only affects the respiratory system, but may also affect the circulatory, digestive, and urinary systems [5]. TMPRSS2 is highly expressed in prostate epithelial cells and a minor percentage of prostate club and hillock cells express both ACE-2 and TMPRSS2 [6]. Additionally, prostate adenocarcinoma cells have been reported to have the highest TMPRSS2 expression of all cancers, highlighting the need to further examine the relationship between genitourinary cancer and COVID-19 [7].

The COVID-19 pandemic is also directly impacting the practice of urology, with many urologists being redeployed to care for infected patients and urology units around the world have had to suspend outpatient activity and limit elective urologic surgery [8].

Treatment decisions for both early and advanced GU (Genito-Urinary) malignancies take into account the risk of dying from the malignancy as well as the risk of death due to other causes such as other co-morbidities. COVID-19 is a new additional and immediate risk to patient’s morbidity and mortality and there is a need for an accurate assessment as to the potential impact on of this syndrome on GU cancer patients.

Urologists and GU oncologists will encounter and care for many patients who are at risk of adverse outcomes; including intubation and death. However, identifying these patients early remains a challenge. A method of predicting a patient’s risk with a high predictive accuracy to assess each patient’s risk of adverse outcomes would be of great value to those involved in the care of patients in this at-risk group. The aim of this study was to construct such a risk tool, identifying those GU cancer patients at highest risk of a positive test result for SARS-CoV-2 infection, hospitalization, intubation, and death.

## 2. Methods

### 2.1. Patient Data

Symptomatic patients presenting to the Mount Sinai Healthcare System (MSHS) who underwent testing for SARS-CoV-2 were included in this dataset (*n* = 38,324). This includes retrospective data from 10 institutes and facilities across 4 boroughs of New York City. The cut-off date for this study was May 26th, 2020. There were 10,362 who tested positive on a reverse transcriptase PCR assay for COVID-19 following a naso-pharyneal swab. The demographic and clinical variables collected included age, sex, race, BMI, reported maximum temperature, temperature at time of review, systolic blood pressure, respiratory rate, lowest oxygen saturation on admission, smoking status, and comorbidities such as asthma, COPD, hypertension, and diabetes.

The MSHS Ethics Committee approved a waiver of documentation of informed consent; de-identified patient data was obtained from the MSHS Data Warehouse (https://msdw.mountsinai.org/).

### 2.2. Statistical Analysis

All analyses were performed using R statistical software [9]. Continuous data were presented as median (interquartile range [IQR]) and means were compared using independent group t-tests for normally distributed data, while non-normally distributed data were assessed using the Mann–Whitney test. Categorical data were presented as number (percentage). The χ^2^ test was used to compare differences in clinical outcomes between groups; COVID test positive and COVID test negative, hospitalized vs. not, intubated vs. not, and deceased vs. alive. The dataset was randomly divided with 80% used to develop the prediction model, and 20% maintained as a holdout dataset. Correlation of available demographic and clinical covariates was performed. Using the available demographic and clinical covariates, and an undisclosed proprietary prediction platform which also captures non-linear interactions, we developed a model to predict a COVID-19 mortality test in the training set, which also used an iterative multivariate analysis strategy to select a subset of the available predictors. This produced multiple models which were then validated in the holdout data set. The model which required the least covariates and which was not statistically significant from the model with the greatest discriminative ability in the holdout dataset was chosen [10].

We assessed the discrimination of the models with the area under the receiver operating characteristic curve (AUC [95% CI]). AUC values for the various models were compared using U-statistics [11]. Calibration curves were used to assess the calibration of the respective models and were computed by comparing observed proportions of a positive COVID-19 test to mean calculated risks from the model in the holdout cohort [12]. Decision curve analysis was performed to assess for the gain derived from using this model in the holdout cohort over the corresponding net benefit curves of testing all of these patients, or none of these patients [13].

## 3. Results

Of the multi-ethnic cohort of 38,324 patients tested for SARS-CoV-2; 880 (2.3%) had a documented GU malignancy and 10,362 (27%) tested positive. Of the 880 patients with a GU malignancy, 484 (55%) had complete clinical and demographic data available; with 149 (30.8%) testing positive for COVID-19. The demographic and clinical characteristics of this cohort is shown in Table 1. Prostate cancer patients accounted for the majority of these GU cancers; 315 had prostate cancer (34.3% of whom tested positive), 113 had bladder cancer, with 25 (22.1%) testing positive. Sixty-nine had kidney cancer, with 21 (30.4%) testing positive. Seventeen had testicular cancer, with 3 (17.6%) testing positive. From this cohort of GU cancer patients, significantly more tested positive for SARS-CoV-2 among those with a diagnosis of prostate cancer, than those with other GU cancers. One hundred and eight of 149 (72.5%) of those from this cohort who tested positive had a diagnosis of prostate cancer, and those with a diagnosis of prostate cancer had a statistically significantly higher risk of testing positive than all other non-prostate GU cancers in this cohort (*p* = 0.0297, see Table 1). Of the 149 patients testing positive for COVID-19, 122 (82%) required hospitalization, 19 (12.8%) required intubation, and 35 (23.5%) died of the disease.

Using demographic and clinical data, the described iterative method employing machine learning methods to predict the outcomes of a positive diagnosis, hospitalization, intubation, and death in the randomly selected test set of patients (comprised of 80% of the cohort), identified age, sex, temperature on admission, systolic blood pressure on admission, respiratory rate on admission, and lowest oxygen saturation on admission as the optimal set of predictors in this cohort.

**Figure 1:**
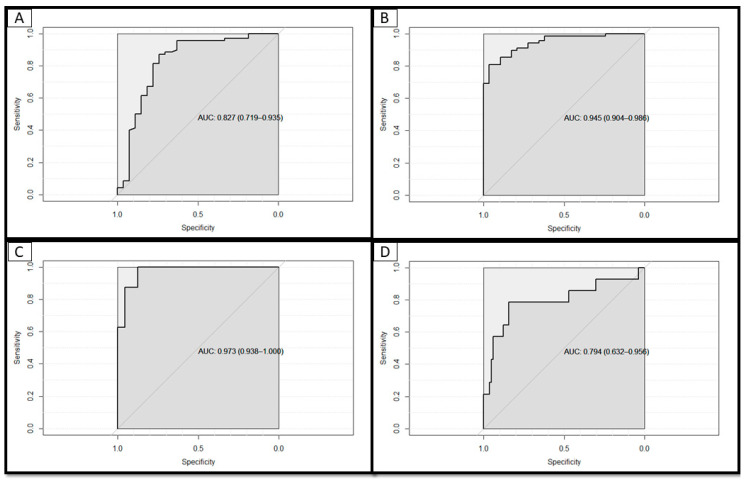
Discriminative ability of each model in the hold-out dataset of patients with GU cancer (*N* = 97) to predict: (**A**) A positive SARS-CoV-2 test, (**B**) hospitalization, (**C**) intubation, and (**D**) death. The diagonal line at 45° represents the performance of a coin toss in discerning the respective outcome, from a non-event. The developed risk tools have a good discriminative ability demonstrating an AUC of 0.83 (95% CI, 0.72–0.94) for the prediction of a positive SARS-CoV-2 test (**A**), 0.95 (95% CI, 0.90–0.99) for predicting the risk of hospitalization (**B**), 0.97 (95% CI, 0.94–1) for the prediction of intubation and 0.79 (95% CI, 0.63–0.96) for the prediction of death in patients in this cohort.

The risk tool for identifying patients at risk of having a positive COVID-19 test, hospitalization, intubation, and death had AUC values of 0.82 (95% CI, 0.72–0.94), 0.95 (95% CI, 0.90–0.99), 0.97 (95% CI, 0.94–1), and 0.79 (95% CI, 0.63–0.96), respectively, in the independent hold-out dataset, suggesting that each model had good discriminative ability (Figure 1).

**Figure 2:**
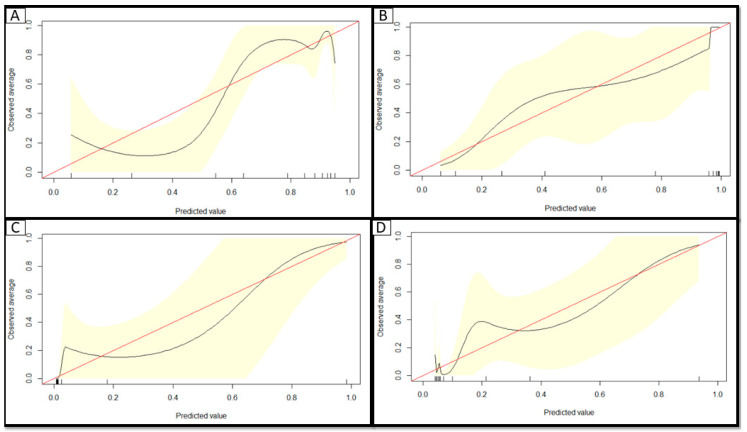
Calibration curves of each model in the hold-out dataset of patients with GU cancer (*N* = 97) to demonstrate the reliability of each developed risk tool to predict: (**A**) A positive SARS-CoV-2 test, (**B**) hospitalization, (**C**) intubation, and (**D**) death. The diagonal line at 45° represents perfect calibration: when the predicted probability of an event perfectly matches the proportion of observed events; as the outcomes here are 0 and 1; Loess smoothing was used to estimate the observed probabilities of the outcome in relation to the predicted probabilities. The developed risk tools generally have an acceptable reliability as assessed by calibration plots: where there is over prediction of a positive SARS-CoV-2 test at predicted values <50% (**A**) and the risk of intubation (**C**) while the models to predict hospitalization and death are well calibrated in this hold-out data subset of this cohort.

Calibration plots provide a visual representation of how reliable the predicted risk estimate is: The accuracy of risk estimates relating to the agreement between estimated and observed events [14]. A curve close to the diagonal indicates that predicted risks correspond well to observed proportions; Figure 2 demonstrates that there is over-prediction of risk of a positive test in those with a predicted risk value of ~<50%, but there is good agreement between observed and predicted risks in the models to predict hospitalization, intubation, and death.

**Figure 3:**
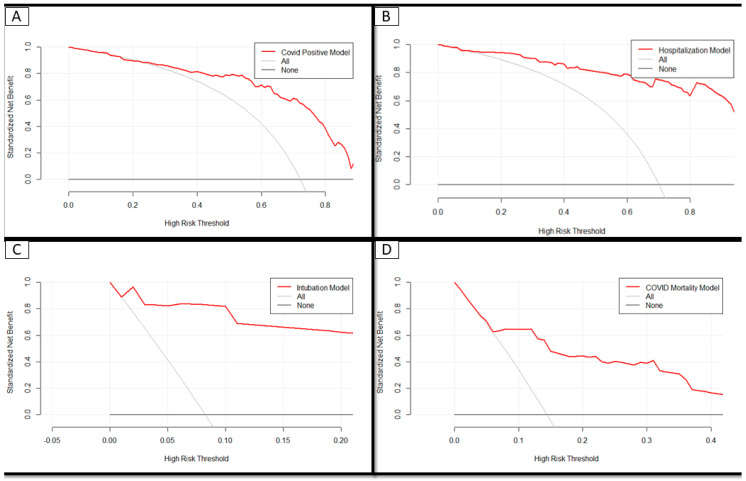
Decision curve analysis of each model demonstrating the superior net benefit of each over other strategies in predicting (**A**) a positive SARS-CoV-2 test, (**B**) hospitalization, (**C**) intubation, and (**D**) death. The horizontal black line at *Y* = 0 represents the net benefit of doing nothing, the curved grey line represents the net benefit of treating everyone while the red line represents the net benefit derived from the a strategy of employing the respective risk calculators.

Decision curve analysis was performed to evaluate the clinical relevance of these models (Figure 3). It is based on the principle that the probability at which a physician would advise treatment is informative on how the physician and patient weigh the harms of false-positive results in comparison with the harms of false-negative results [12]. This probability is referred to as the threshold probability. This threshold probability can then be used to derive the net benefit of the model across different threshold probabilities: The straight black line at *y* = 0 represents the net benefit derived from employing a strategy of testing nobody and the grey line represents the net benefit if a strategy of testing everybody was employed. Each model was superior to both of these strategies across the entire range of clinically useful threshold risks.

## 4. Discussion

Treatment decisions for both early and advanced GU malignancies take into account the risk of dying from the malignancy as well as the risk of death due to other causes such as other co-morbidities. COVID-19 is a new additional and immediate risk to patient’s morbidity and mortality and there is a need for an accurate assessment as to the potential impact on of this syndrome on GU cancer patients.

To our knowledge, this is the first risk tool developed to identify those most at risk of intubation and death from COVID-19 in the at-risk subgroup of those with a GU malignancy.

Cancer patients have a higher susceptibility to acquiring an infection due to their immunocompromised state as compared to the general population, and specifically for COVID-19 they have over a 3-fold increase in the risk of a serious event [15]. They have a higher risk of intensive care unit (ICU) admission, intubation, and death [16]. GU cancers accounted for 14.5% of cancers in our cohort of 38,324 patients assessed, which is similar to previously published figures of ~15%, and cancer care is a significant workload in all urology departments [8]. There are numerous publications now in literature which make various recommendations on which uro-oncology surgeries can proceed or be deferred during this pandemic [17]. To our knowledge, this work is the first to accurately identify those uro-oncology patients at risk of being diagnosed with COVID-19, their risk of hospitalization, intubation and death from COVID-19.

Our risk tool uses readily available demographic and clinical data from a multi-ethnic cohort to predict the likelihood of significant events in this at-risk population. Other studies assessing the risk of severe COVID-19 disease (defined based upon clinical and laboratory criteria) in ethnically homogenous cohorts have identified factors such as older age, LDH, CRP, RDW and direct bilirubin (DBIL), and blood urea nitrogen (BUN); and lower albumin (ALB) on admission, temperature, cough, dyspnea, hypertension, cardiovascular disease, chronic liver disease, and chronic kidney disease [18,19].

The discriminative ability of our risk tool (0.97 for predicting the risk of intubation in GU cancer patients, and 0.79 for predicting the risk of death) compares favorably with that of other similar risk tools and nomograms for COVID-19 in the general population. Gong et al. obtained an AUC value of 0.853 in their validation cohort for predicting severe COVID-19 disease and Zhou et al. obtained an AUC of 0.839 in their bootstrapped analysis for predicting severe COVID-19 disease; both using a definition severe disease from the Chinese CDC which was defined as the patient developing any of the following: (1) shortness of breath (respiratory rate ≥30 breaths per min), (2) oxygen saturation ≤93% at rest, or (3) arterial partial pressure of oxygen/fraction of inspired oxygen ≤300 mm Hg [18,19]. The endpoints we employed in this study are of greater utility though as they predict patient outcomes as opposed to measure of a vital sign or arterial partial pressure of oxygen.

Limitations to our work include that this is a retrospective study including 484 patients with prostate, bladder, kidney, or testicular cancer being assessed for SARS-CoV-2 infection at an MSHS facility across New York. Due to the deidentified nature of the dataset, we do not have data on cancer staging or details of specific cancer therapies patients have received. However, it is a relatively unique dataset given its overall size, multi-ethnic composition, and diversity of social groups represented, and the proportion of GU-cancers amongst the cohort is similar to previously published figures of incidence. Given the federal mandate in the USA that all COVID-19 testing and treatment is free, one would expect that this cohort is not exclusive of those uninsured patients, however this explicit data point is not available to us.

## 5. Conclusions

Recent publications have discussed the use of machine learning and artificial intelligence in medicine as “a fundamental technology required to meaningfully process data” [20]. We believe there is an unprecedented opportunity for machine learning driven prediction models to inform, personalize and improve care.

We have developed a novel risk model which can predict diagnosis, hospitalization, intubation, and mortality for patients with GU cancers and COVID-19 which is both accurate and reliable. This freely available online risk tool can aid clinicians to identify those GU cancer patients at risk of intubation and death.

The clinical application of this risk tool and other predictive models have the potential to aid urologists in risk stratification of their patients for the diagnosis of COVID-19 and its complications such as admission, intubation, and death. For urology departments that are continuing to perform urgent elective care of cancer patients, this risk tool could aid in the decision-making process to risk stratify those patients at greatest risk of COVID-19-related adverse outcomes. A better understanding of which patient dies, and furthermore which patient is in most critical need of care, could help better inform our understanding of the COVID-19 syndrome and better care for all patients.

## Figures and Tables

**Table 1:** Demographic and clinical characteristics of the entire cohort (*N* = 484).

	Not Detected	Positive	*p*-Value
	(*N* = 335)	(*N* = 149)	
**Age (years)**			
Mean (SD)	69.0 (14.6)	71.8 (10.6)	0.0169
**Sex**			
Male (%)	299 (89.3%)	139 (93.3%)	
**Race/ethnicity**			
African ancestry	92 (27.5%)	41 (27.5%)	<0.001
Asian	8 (2.4%)	4 (2.7%)	
Hispanic/Latinx	48 (14.3%)	45 (30.2%)	
Other/unknown	46 (13.7%)	21 (14.1%)	
White	141 (42.1%)	38 (25.5%)	
**English as preferred language**			
Yes (%)	303 (90.4%)	117 (78.5%)	<0.001
**Smoking status**			
Never	119 (35.5%)	71 (47.7%)	0.00347
Not asked	11 (3.3%)	6 (4.0%)	
Quit	162 (48.4%)	67 (45.0%)	
Yes	43 (12.8%)	5 (3.4%)	
**Asthma**	17 (5.1%)	8 (5.4%)	1
**COPD**	33 (9.9%)	10 (6.7%)	0.343
**Hypertension**	169 (50.4%)	86 (57.7%)	0.168
**Obesity**	30 (9.0%)	15 (10.1%)	0.826
**Diabetes**	69 (20.6%)	46 (30.9%)	0.0195
**CKD**	56 (16.7%)	31 (20.8%)	0.34
**HIV**	9 (2.7%)	6 (4.0%)	0.616
**Prostate cancer**	207 (61.8%)	108 (72.5%)	0.0297
**Bladder cancer**	88 (26.3%)	25 (16.8%)	0.0306
**Kidney cancer**	48 (14.3%)	21 (14.1%)	1
**Testis cancer**	14 (4.2%)	3 (2.0%)	0.354
**BMI (Kg/m^2^)**			
Mean (SD)	25.7 (5.39)	26.7 (5.44)	0.0591
**Maximum temperature (°F)**			
Mean (SD)	99.5 (1.49)	101 (1.72)	<0.001
**Heart rate (BPM)**			
Mean (SD)	91.4 (22.4)	94.7 (19.5)	0.1
**Respiratory rate (breaths/minute)**			
Mean (SD)	18.8 (2.82)	20.0 (4.85)	0.0048
**Systolic BP (mm/Hg)**			
Mean (SD)	133 (25.4)	129 (23.3)	0.123
**Diastolic BP (mm/Hg)**			
Mean (SD)	76.0 (15.5)	72.7 (14.0)	0.0219
**Minimum o2 saturation (%)**			
Mean (SD)	91.6 (12.7)	84.2 (17.7)	<0.001
**Intubated**	16 (4.8%)	19 (12.8%)	0.00331
**Hospitalized**	201 (60.0%)	122 (81.9%)	<0.001
**Deceased**	29 (8.7%)	35 (23.5%)	<0.001

COPD = Chronic obstructive pulmonary disease. CKD = chronic kidney disease. BMI = body mass index.

## Data Availability

Data managed by msdw.mountsinai.org.
